# Novel Protonic Conductor SrLa_2_Sc_2_O_7_ with Layered Structure for Electrochemical Devices

**DOI:** 10.3390/ma15248867

**Published:** 2022-12-12

**Authors:** Nataliia Tarasova, Anzhelika Bedarkova, Irina Animitsa, Ekaterina Abakumova, Vladislava Gnatyuk, Inna Zvonareva

**Affiliations:** 1 The Institute of High Temperature Electrochemistry of the Ural Branch of the Russian Academy of Sciences, 620002 Yekaterinburg, Russia; 2 Institute of Hydrogen Energy, Ural Federal University, 620002 Yekaterinburg, Russia

**Keywords:** hydrogen energy, layered perovskite, proton conductivity

## Abstract

Novel materials with target properties for different electrochemical energy conversion and storage devices are currently being actively created and investigated. Materials with high level of protonic conductivity are attracting attention as electrolytes for solid oxide fuel cells and electrolyzers. Though many materials are being investigated as potential electrolytic components for these devices, many problems exist, including comparability between electrodes and electrolytes. In this paper, layered perovskite SrLa_2_Sc_2_O_7_ was investigated as a protonic conductor for the first time. The possibility for water uptake and protonic transport was revealed. It was shown that the SrLa_2_Sc_2_O_7_ composition can be considered a prospective ionic conductor. The layered perovskites can be considered as very promising materials for electrochemical devices for energy applications.

## 1. Introduction

Novel materials with target properties for different electrochemical energy conversion and storage devices are currently being actively created and investigated [1,2,3,4]. These devices must meet certain requirements, such as high effectiveness, low cost, eco-friendliness and safety. Hydrogen energy satisfies those criteria well, and can be considered one of the most promising energy sources for the future [5,6,7,8,9]. Accordingly, the development of systems for the production, transportation and conversion of hydrogen is necessary. Protonic ceramic fuel cells are electrochemical devices that convert the chemical energy of hydrogen oxidation into electrical energy. The main components of such devices are electrolytes [10,11,12,13,14] and electrodes [15,16]. Though many materials have been investigated as potential electrolytic and electrode components for these devices, many problems exist, including comparability between electrodes and electrolytes [17,18,19,20]. The most studied proton-conducting materials for use as electrolytes in protonic ceramic fuel cells are barium cerate-zirconates BaCeO_3_–BaZrO_3_, which are characterized by a perovskite structure [21,22]. However, promising electrode materials such as nickelites [23,24,25,26] and cobaltates [27,28,29] have layered perovskite structure. Consequently, the creation of proton-conductive materials with layered perovskite structure is very important from the point of view of comparability between electrolyte and electrode materials.

Layered perovskites can be described by the general formula AA’_n_B_n_O_3n+1_, where A is the alkali-earth metal, such as barium or strontium, A’ is the rare-earth metal, such as lanthanum or neodymium, and B is the trivalent metal, such as indium or scandium. Monolayer perovskites AA’BO_4_ (n = 1) were described as protonic conductors several years ago for the first time [30]. Such matrix compositions as BaNdInO_4_ [31,32,33,34,35], BaNdScO_4_ [36], SrLaInO_4_ [37,38,39,40,41], BaLaInO_4_ [42,43,44,45,46,47] and compounds based on them were investigated, and general regularities of proton transport in doped monolayer perovskites were revealed [48]. Two-layer perovskites with the general formula AA’_2_B_2_O_7_ (n = 2), such as BaLa_2_In_2_O_7_ [49,50,51,52] and BaNd_2_In_2_O_7_ [53], were described as proton-conducting materials earlier this year. It was proven that they are nearly pure protonic conductors below 350 °C in wet air. Accordingly, two-layer perovskites are a promising class of materials in terms of their protonic conductivity. In this paper, layered perovskite SrLa_2_Sc_2_O_7_ was investigated as a protonic conductor for the first time. The local structure, possibility for water uptake and protonic transport were revealed.

## 2. Materials and Methods

Composition SrLa_2_Sc_2_O_7_ was synthesized using a solid-state method. The starting reagents SrCO_3_, La_2_O_3_ and Sc_2_O_3_ (for all 99.99% purity, REACHIM, Moscow, Russia) were used. The final temperature of calcination was 1300 °C.

The XRD investigations were performed using a Bruker Advance D8 Cu K*_α_* diffractometer (step of 0.01°, scanning rate of 0.5°/min, Bruker, Billerica, MA, USA). Raman spectra were collected on the modular confocal Raman microscopy system Alpha 300 AR (WiTec, Ulm, Germany). The 10× objective lens (numerical aperture 0.2) were used to the focus the blue laser (l = 488 nm, averaging three spectra) to a spot size around 3 μm. The morphology and chemical composition of the samples were studied using a VEGA3 TESCAN scanning electron microscope (SEM, TESCAN, Brno, Czech Republic) equipped with a system for energy-dispersive X-ray spectroscopy (EDS).

The thermogravimetry (TG) was made using an STA 409 PC Netzsch Analyser (NETZSCH, Selb, Germany). The heating of the initially hydrated samples was made at the temperature range of 40–1100 °C with the rate of 10 °C/min under a flow of dry Ar.

The electrical conductivity was measured using impedance spectrometer Z-1000P, Elins, Chernogolovka, Russian. The investigations were made from 1000 to 200 °C with 1°/min cooling rate under dry air or dry Ar conditions. The dry gas (air or Ar) was produced by circulating the gas through P_2_O_5_ (*p*H_2_O = 3.5 × 10^−5^ atm). The wet gas (air or Ar) was obtained by first bubbling the gas at room temperature through distilled water and then through a saturated solution of KBr (*p*H_2_O = 2 × 10^−2^ atm).

## 3. Results

**Figure 1:**
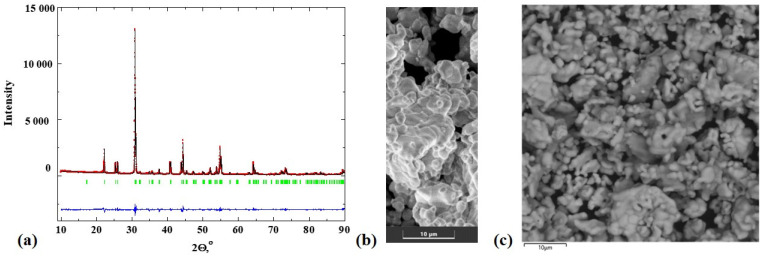
The XRD-patterns (**a**) (R_p_ = 1.99, R_wp_ = 2.03, χ^2^ = 1.09) and SEM-image (**b**,**c**) of powder sample SrLa_2_Sc_2_O_7_.

Figure 1a represents the results of the XRD-analysis for the obtained SrLa_2_Sc_2_O_7_ composition. All peaks correspond to the *Fmmm* space group, and their calculated lattice parameters (Table 1) are well correlated with previously reported data [54,55] (ICSD 67625). Figure 1b represents the results of SEM investigations. The SrLa_2_Sc_2_O_7_ compositions consists of agglomerates (~10−20 μm) of grains (~3−5 μm) with irregular shape.

The elements ratio was determined using EDS analysis. The average element ratios determined by EDS analysis for the SrLa_2_Sc_2_O_7_ compositions were 8.1 (8.3) for Sr, 16.6 (16.7) for La, 16.8 (16.7) for Sc and 58.5 (58.3) for O, where theoretical values are in brackets. A good agreement between the theoretical and experimental values was confirmed.

**Figure 2:**
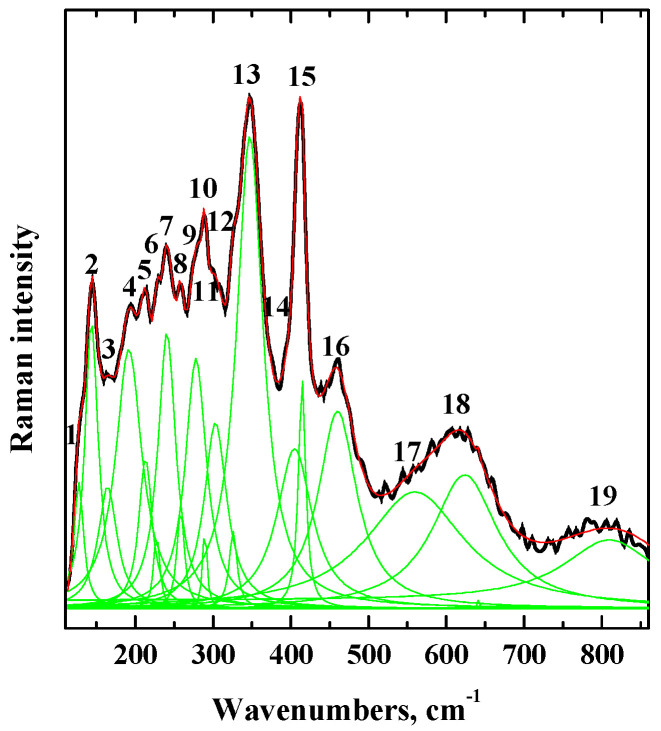
Raman spectrum of SrLa_2_Sc_2_O_7_ composition.

Local structure of the SrLa_2_Sc_2_O_7_ composition was investigated using the Raman spectroscopy method. Figure 2 represents the deconvolution of the Raman spectrum for the SrLa_2_Sc_2_O_7_ composition.

The low-wavenumbers region (120−200 cm^−1^) contains several signals corresponded to the stretching and bending vibrations of alkali-earth- and rare-earth-containing metal polyhedra [51,56,57,58,59] (Table 2). The tilting/bending and stretching vibrations of trivalent metal with small ionic radii polyhedra (scandium, in our case [60]) should be located in the mid- and high-wavenumbers region (higher 200 cm^−1^). This region contains more signals as compared to BaLa_2_In_2_O_7_ [53], which can indicate an increase in the deformation of polyhedra [ScO_6_] in the structure of SrLa_2_Sc_2_O_7_ compared with polyhedra [InO_6_] in the structure of BaLa_2_In_2_O_7_. The signals in the 500−900 cm^−1^ wavenumbers region correspond to the repulsion between the Sr^2+^/La^3+^ ions and oxygen ions in compressed Sc-contained polyhedra [61], i.e., it proves the deformation of Sc-contained polyhedra. The additional confirmation of this is the decrease in the lattice parameters and unit cell volume in the row BaLa_2_In_2_O_7_−SrLa_2_Sc_2_O_7_ (Table 1).

**Figure 3:**
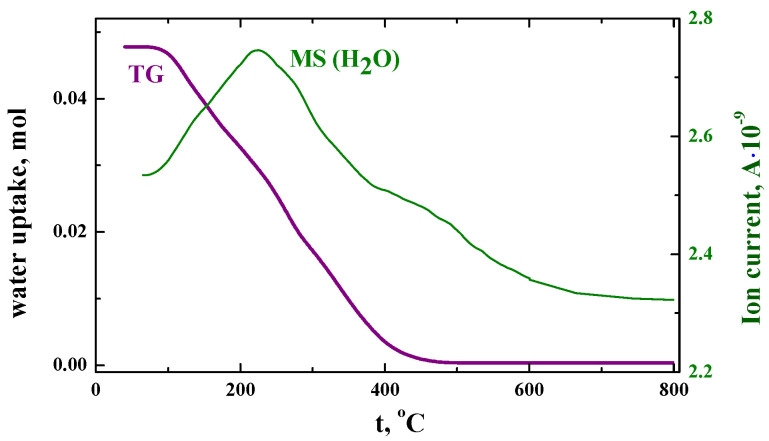
TG- and MS(H_2_O)-results for hydrated SrLa_2_Sc_2_O_7_ composition.

The possibility of interaction of the investigated composition with water vapors was checked using thermogravimetry (TG) measurements (Figure 3). As can be seen, the SrLa_2_Sc_2_O_7_ composition can dissociatively intercalate some amount of water molecules, however, the water uptake is not much; about 0.05 mol H_2_O per mol complex oxide. The mass spectroscopy (MS) results confirm the release of water during heating. At the same time, water uptake for the BaLa_2_In_2_O_7_ composition was about 0.17 mol H_2_O per formula unit [53]. For the layered perovskites, the possibility of water uptake is due to the presence of enough space between the perovskite blocks and the rock-salt layers [48]:

(1) $$H_{2}O+O_{o}^{x}\Leftrightarrow {(\mathrm{OH})}_{o}^{•}+{\left(\mathrm{OH}\right)}_{i}^{\prime }$$

where ${(\mathrm{OH})}_{o}^{•}$ is the hydroxyl group in the regular oxygen position; ${\left(\mathrm{OH}\right)}_{i}^{\prime }$ is the hydroxyl group located in the interlayer space. The increase of the size of this space leads to the increase of the water uptake. Accordingly, the decrease of the unit cell volume and the decrease of this space should lead to a decrease of the water uptake. In other words, the concentration of protons decreases in the BaLa_2_In_2_O_7_−SrLa_2_Sc_2_O_7_ row in accordance with the decrease of the unit cell volumes (Table 1).

**Figure 4:**
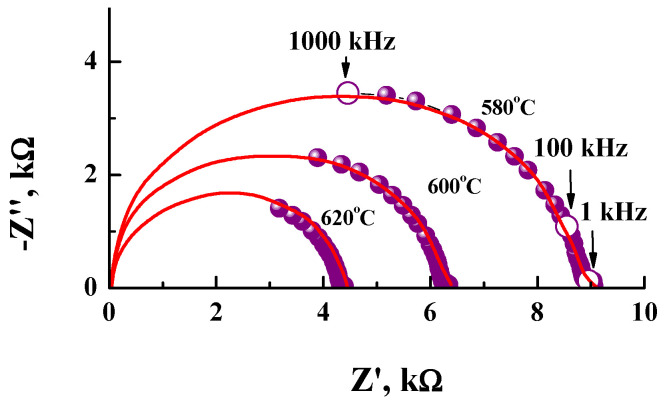
EIS plots for SrLa_2_Sc_2_O_7_ composition obtained under dry air at 580, 600 and 620 °C.

The electrical conductivity values were collected using the impedance spectroscopy method. The EIS plots for the SrLa_2_Sc_2_O_7_ composition obtained at different temperatures are presented in Figure 4. All EIS plots consist of two semicircles. The fitting of the spectra was made using ZView software (Scribner, Southern Pines, NC, USA), and the obtained results are presented in Table 3. The first (high-frequency) corresponds to volume resistance and has a capacitance of ~10^−12^ F/cm. The second semicircle (very small) corresponds to grain boundaries resistance and has a capacitance of ~10^−10^ F/cm. To calculate conductivity, we used the resistance value of the sample obtained by extrapolating the high-frequency semicircle to the abscissa axis (approximation with using the Zview software).

**Figure 5:**
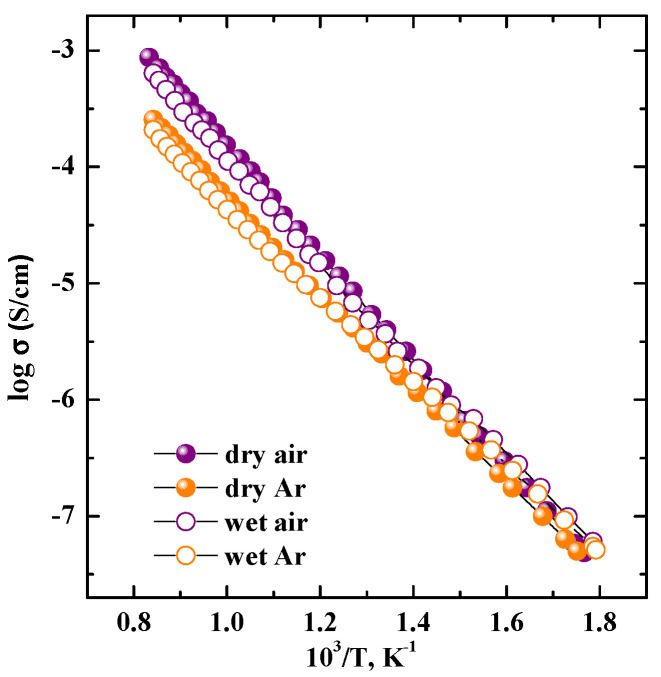
Temperature dependencies of conductivity for SrLa_2_Sc_2_O_7_ composition obtained under dry (filled symbols) and wet (open symbols) conditions.

The temperature dependencies of conductivity are presented in Figure 5. The conductivity values obtained under high temperatures and dry air conditions (pO_2_ = 0.21 atm) are higher than those obtained under dry Ar conditions (pO_2_~10^−5^ atm, conditions of dominance of oxygen-ionic conductivity), which confirms the mixed oxygen–hole nature of conductivity:

(2) Vo••+12O2⇔Oox+2h•

where $V_{O}^{••}$ is the oxygen vacancy; $h^{•}$ is the hole. However, the temperature decreasing leads to the increase in the oxygen transport share from 30% at 900 °C to 70% at 300 °C. It should be noted that the BaLa_2_In_2_O_7_ composition is characterized by mixed oxygen–hole conductivity, with a 20% share of oxygen transport in the entire temperature 900−300 region [53].

**Figure 6:**
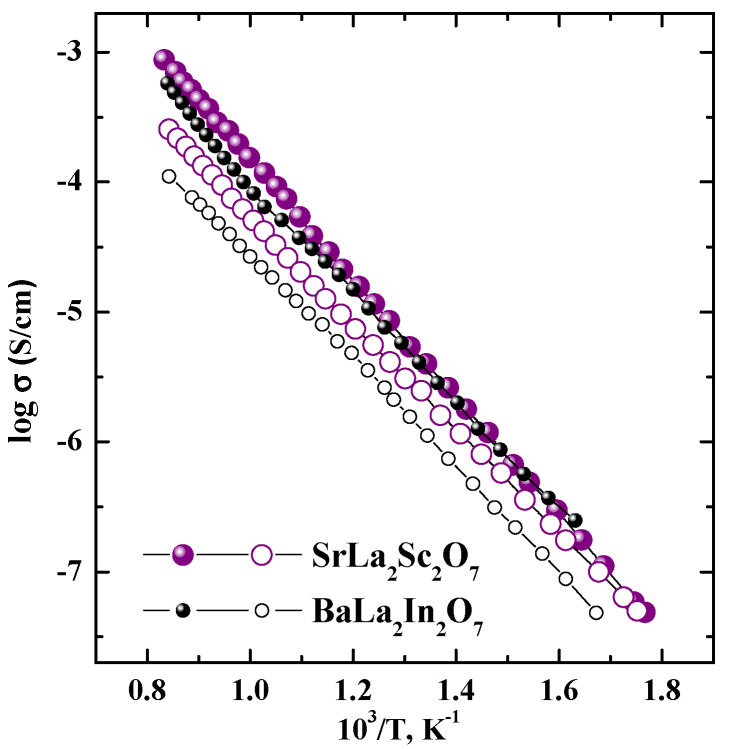
Temperature dependencies of conductivity for SrLa_2_Sc_2_O_7_ and BaLa_2_In_2_O_7_ compositions obtained under dry air (filled symbols) and dry Ar (open symbols) conditions.

Figure 6 represents the comparison of the temperature dependencies for the SrLa_2_Sc_2_O_7_ and BaLa_2_In_2_O_7_ compositions obtained under dry conditions. As can be seen, the conductivity values obtained under dry air are close, and the conductivity values obtained under dry Ar (oxygen-ion conductivity) are higher for the SrLa_2_Sc_2_O_7_ composition. In other words, the SrLa_2_Sc_2_O_7_ composition is more preferable from the point of view of oxygen–ionic conductivity compared with the BaLa_2_In_2_O_7_ composition.

**Figure 7:**
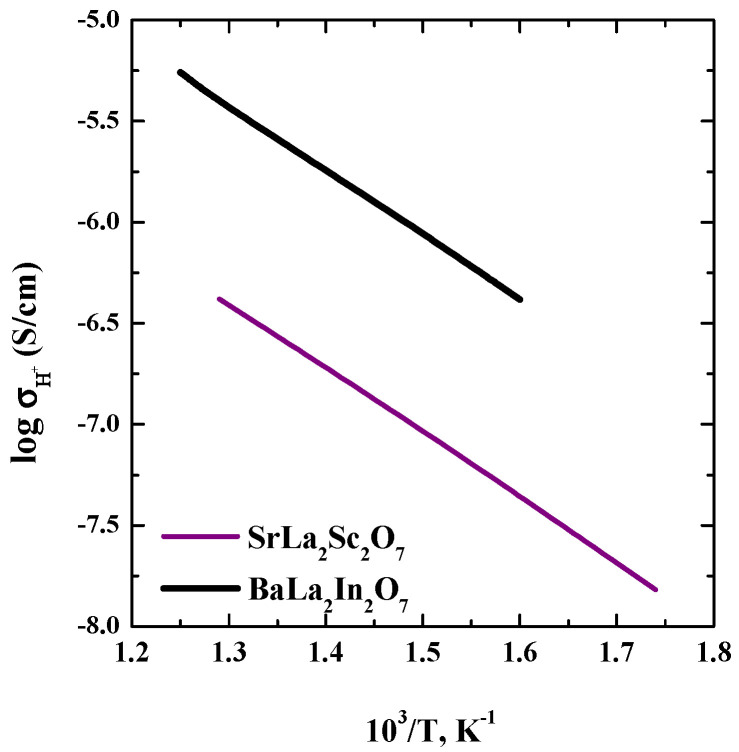
Temperature dependencies of protonic conductivity for SrLa_2_Sc_2_O_7_ and BaLa_2_In_2_O_7_ composition.

The conductivity values obtained for the SrLa_2_Sc_2_O_7_ composition under wet conditions are presented in Figure 5 (open symbols). The effect of humidity on the conductivity values started lower, at ~400 °C, which correlates well with TG-data. At the same time, the conductivity values obtained under wet air and wet Ar below 400 °C are very close, which indicates the ionic (protonic) nature of conductivity under wet air conditions and low temperatures. The proton conductivity was calculated as the difference between the conductivity values obtained under wet Ar and dry Ar, i.e., as:

(3) $$\sigma_{H^{+}}=\sigma_{wet\;Ar}-\sigma_{dry\;Ar}=\sigma_{wet}^{ion}-\sigma_{dry}^{ion}$$

and its temperature dependences are shown in Figure 7. The calculation was made for the temperatures 300, 350, 400, 450 and 500 °C.

As can be seen, the protonic conductivity values for the SrLa_2_Sc_2_O_7_ composition are lower than for the BaLa_2_In_2_O_7_ composition. It is clear that this decrease is due to a decrease of the proton concentration for SrLa_2_Sc_2_O_7_ compared with BaLa_2_In_2_O_7_. Meanwhile, the SrLa_2_Sc_2_O_7_ composition is very promising prospective ionic conductor. The increase of the unit cell volume by the doping, for example, can lead to an increase of the proton concentration in the structure and an increase of the proton conductivity.

## 4. Conclusions

The layered perovskite SrLa_2_Sc_2_O_7_ was investigated as a protonic conductor for the first time. The local structure, possibility for water uptake and protonic transport was revealed. The doping of the layered perovskite structure potentially can increase the proton conductivity. Based on this, the layered perovskite SrLa_2_Sc_2_O_7_ can be considered as a very promising material for energy applications in electrochemical devices.

## Figures and Tables

**Table 1:** The lattice parameters and unit cell volumes of the compositions SrLa_2_Sc_2_O_7_ and BaLa_2_In_2_O_7_.

Composition	*a,* Å	*b,* Å	*c,* Å	Unit Cell Volume, (Å^3^)
SrLa_2_Sc_2_O_7_	5.781(1)	5.738(1)	20.534(2)	681.17(1)
SrLa_2_Sc_2_O_7_ [54]	5.781(8)	5.736(7)	20.534(2)	681.09(9)
SrLa_2_Sc_2_O_7_ [55]	5.781(8)	5.736(7)	20.534(2)	681.08(7)
BaLa_2_In_2_O_7_ [49]	5.914(9)	5.914(9)	20.846(5)	729.33(6)

**Table 2:** Wavenumbers (cm^−1^) of Raman bands for the SrLa_2_Sc_2_O_7_ compound.

No of Band	Wavenumber, cm^−1^
1	128
2	144
3	165
4	191
5	214
6	228
7	240
8	257
9	278
10	289
11	303
12	326
13	348
14	406
15	416
16	461
17	560
18	625
19	810

**Table 3:** Results of EIS plots fitting, where CPE is the constant phase element (F), and R is the resistance (kΩ∙cm).

Element	Value (580 °C)	Value (600 °C)	Value (620 °C)
CPE1	1.9 × 10^−12^	2.1 × 10^−12^	2.2 × 10^−12^
R1	8.3	5.8	3.9
CPE2	3.1 × 10^−10^	3.5 × 10^−10^	2.0 × 10^−10^
R2	9.2	6.5	4.5

## Data Availability

Not applicable.
